# Activation of Gcn2 in response to different stresses

**DOI:** 10.1371/journal.pone.0182143

**Published:** 2017-08-03

**Authors:** Silje Anda, Róbert Zach, Beáta Grallert

**Affiliations:** Department of Radiation Biology, Institute for Cancer Research, Oslo University Hospital, Oslo, Norway; University of Cambridge, UNITED KINGDOM

## Abstract

All organisms have evolved pathways to respond to different forms of cellular stress. The Gcn2 kinase is best known as a regulator of translation initiation in response to starvation for amino acids. Work in budding yeast has showed that the molecular mechanism of GCN2 activation involves the binding of uncharged tRNAs, which results in a conformational change and GCN2 activation. This pathway requires GCN1, which ensures delivery of the uncharged tRNA onto GCN2. However, Gcn2 is activated by a number of other stresses which do not obviously involve accumulation of uncharged tRNAs, raising the question how Gcn2 is activated under these conditions. Here we investigate the requirement for ongoing translation and tRNA binding for Gcn2 activation after different stresses in fission yeast. We find that mutating the tRNA-binding site on Gcn2 or deleting Gcn1 abolishes Gcn2 activation under all the investigated conditions. These results suggest that tRNA binding to Gcn2 is required for Gcn2 activation not only in response to starvation but also after UV irradiation and oxidative stress.

## Introduction

All cells and organisms are surrounded by a changing and often stressful environment and have developed various signaling pathways to adapt to these changes. An important requirement for maintaining cell homeostasis during stress conditions is the correct regulation of translation. Translational regulation in response to different types of stress involves phosphorylation of serine51 (Ser 52 in *S*. *pombe*) of the eukaryotic translation initiation factor 2α (eIF2α) [1]. This phosphorylation is thought to lead to a general downregulation of translation, accompanied by an enhanced translation of specific stress-response mRNAs [2–4]. One of the eIF2α kinases performing this phosphorylation is Gcn2. Gcn2 was first described in budding yeast as a regulator of eIF2α phosphorylation in response to amino-acid starvation. This role is conserved from yeast to human cells and the extent of conservation is such that the human Gcn2 can functionally replace the budding yeast Gcn2 [5]. Fission yeast has several eIF2α kinases and it is GCN2 that is activated in response to nutrient deprivation [6–9].

The mechanism of GCN2 activation in response to amino-acid starvation has been extensively studied through the years, mainly in budding yeast. Under starvation conditions, uncharged tRNAs accumulate and bind a histidyl-tRNA synthetase-like domain (HisRS) in Gcn2. This, in turn, leads to a conformational change and activates the kinase. This model is supported by the findings that (i) mutations leading to amino-acid substitutions close to the predicted active site of the HisRS domain affect Gcn2 activation, either by leading to constitutive activation or abolishing kinase activation [10–12]; (ii) activating mutations that mimic tRNA binding have been identified [13] and (iii) Gcn2 was shown to be associated with the translating ribosome [14], the very site where the absence of charged tRNA-s is most likely to have an effect.

In budding yeast, the association of GCN2 with the ribosome depends on its interaction with GCN1, a cofactor required for Gcn2 activation upon amino-acid starvation. GCN1 binds the ribosome at or near the A site and is thus perfectly placed to ensure the transfer of the uncharged tRNA onto GCN2 [15–19].

In addition to amino-acid starvation various other types of stress can activate Gcn2, including ultraviolet light (UVC), MMS, H_2_O_2_, proteasome inhibition, viral infections and serum starvation [20,21]. However, it is not immediately obvious how all these different forms of stress might lead to an accumulation of uncharged tRNAs. Furthermore, accumulation of uncharged tRNAs during starvation is a slow process, whereas the response to for example UV and H_2_O_2_ is very fast. Therefore we reasoned that Gcn2 might be activated by other mechanisms.

Here we investigated the mechanism of Gcn2 activation in response to different types of stress. We found that Gcn1 is required for Gcn2 activation after amino-acid starvation also in fission yeast. We show that ongoing translation is not required for UVC-induced activation of Gcn2. However, mutating the tRNA-binding site on Gcn2 or deleting Gcn1 abolishes Gcn2 activation not only in response to starvation but also after UVC irradiation and oxidative stress. These results strongly suggest that tRNA binding is required for Gcn2 activation in response to all these types of stress.

## Materials and methods

### Strains and media

All strains used are derived from the *Schizosaccharomyces pombe* L972h- strain, and are listed in Table 1. The growth conditions and media were as described in [22]. The cells were grown in liquid Edinburgh minimal medium (EMM) containing the required supplements, or yeast extract medium (YES) at 25°C, to a cell density of 3–5 X 10^6^ cells/ml (OD_595_ 0,15–0,3).

**Table 1:** Strains used in this study.

Strain number	Genotype	Source
38	ura4-D18 leu1-32 h+	Paul Nurse
1136	gcn2::ura4+ leu1-32 ura4-D18h-	Ronald Wek
2079	gcn2-F1066A R1067L leu-32 ura4-D18 h-	This work
2095	gcn1::kanMX6 ura4-D18 leu1-32 h+	This work
2120	gcn1::kanMX6 cdc10-M17 mcm2:GFP:kanR ura4-D18 leu1-32	This work

### Leucine starvation

Leucine-auxotroph cells were grown in the presence of leucine in EMM medium and washed by filtering with three volumes of EMM lacking leucine. The cells were then resuspended in EMM lacking leucine and samples were collected at different timepoints after leucine removal.

### Oxidative stress

Cells grown in EMM to a density of 4X 10^6^/ml were treated with H_2_O_2_ at the indicated concentrations, and samples were collected at different timepoints after addition of the oxidative agent.

### UVC-irradiation

Cells were grown in EMM medium to early log phase (OD = 0.15 Fission yeast cells suspended in a thin layer (3 mm) of rapidly stirred liquid EMM medium were irradiated with 254-nm UVC light. The dose was measured with a radiometer (Ultraviolet Products, San Gabriel, CA, USA), and a dose of 1100 J/m^2^ was given at an incident dose rate of approximately 250 J/m^2^/min [23]. This dose gives a survival >90%.

### Pre-RC loading assay

In situ chromatin binding assay was performed as described previously [23,24].

### Immunoblots

Samples for immunoblotting were made by the trichloroacetic acid (TCA) protein extraction method [25]. A total of 50–100 μg protein extracts were run on 10% SDS-PAGE, transferred to a PVDF membrane (Immobilon, EMD Millipore Corporation, Billerica, MA, USA) and probed with the following antibodies: anti-phosphorylated eIF2α (Cat. # 44–728G, Life Technologies, Carlsbad, California, USA) 1:3000; anti-α-tubulin (Cat. # T-5168 Sigma) 1:30 000, anti eIF2α (Cat. # sc-11386, Santa Cruz).

### Translation assay

Cells were pulse-labelled with the methionine analogue L-Homopropargylglycine (HPG; Life Technologies) at a concentration of 50 μM for 10 minutes. Samples were taken at the indicated timepoints after treatment and fixed in ice-cold 70% ethanol. Newly synthesized proteins were detected by chemoselective fluorescence tagging by means of “click chemistry” [26] using the Click-iT Cell reaction buffer kit (Life Technologies) according to the manufacturer’s protocol. The Alexa Fluor 647- specific fluorescence signal was measured by flow cytometry to detect median fluorescence intensity from 10 000 fission yeast cells. Samples without HPG were used as negative controls.

### Gcn2 kinase assays

Gcn2 was immunoprecipitated from exponentially growing unirradiated (“C”) and UV-irradiated “UV” cells, using IP buffer (50 mM Hepes, pH7.5;1 mM EDTA; 20 mM Na-β-glycerolphosphate; 0.1 mM Na3VO4; 50 mM NaF; 75 mM NaCl; 0,1 mM PMSF; 1mM DTT; 0.3% Np40; Roche protease inhibitor cocktail; 1% triton). The immunoprecipiates were resuspended in kinase buffer Tris-HCl pH 7,5; 5 mMMgCl2; 75 mM NaCl DTT 1mM; 0,5% Triton; Protease inhibitor from Roche; 0,1 mM PMSF; 100 μM Na3VO4; 100 μM ATP and mixed with extracts made from an unirradiated culture of *gcn2Δ* cells. After 30 min incubation EDTA was added to 20 mM, Laemmli sample buffer to 1X and the reaction mix was boiled for 5 min before loading on a gel and immunoblotting for eIF2α-P.

## Results

### Ongoing translation is not required for the UVC-induced activation of Gcn2

**Fig 1:**
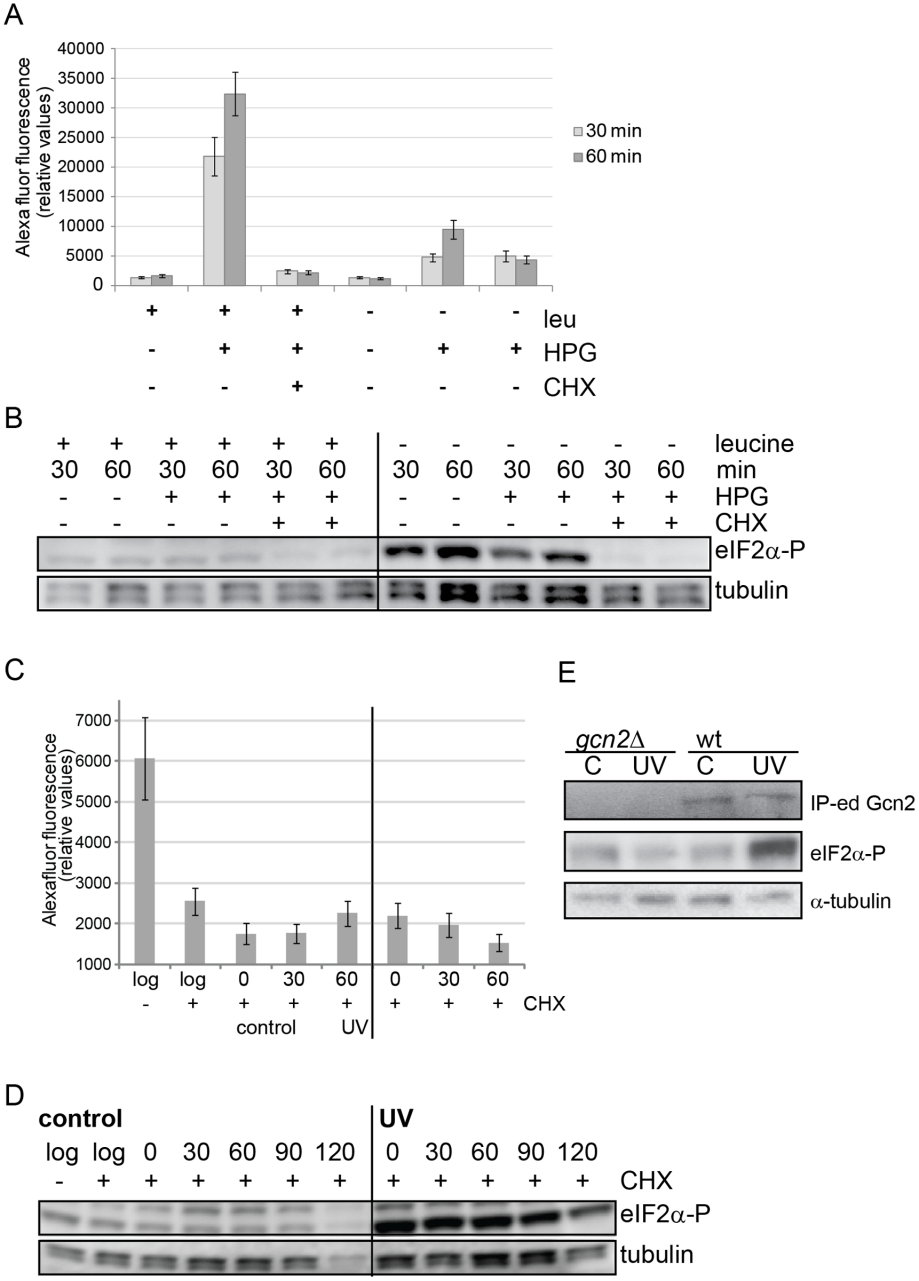
Inhibition of translation does not prevent Gcn2 activation after UV irradiation in fission yeast.

Under starvation conditions uncharged tRNAs accumulate as the existing pool of charged tRNAs is gradually exhausted during protein synthesis. If Gcn2 activation occurs through a similar mechanism of tRNA accumulation also after UV irradiation, one would expect that ongoing translation is also required for activation. To address this, we inhibited translation prior to UVC-irradiation and measured eIF2α phosphorylation to assess Gcn2 activation. To inhibit translation we treated the cells with 100 μg/ml cycloheximide for 10 minutes, under which conditions no further translation could be detected as measured by incorporation of HPG, a methionine analogue (Fig 1A and 1C). To verify that our experimental design can be used to explore the requirement for ongoing translation, we starved fission yeast cells for leucine by withdrawing it from the medium of leucine-auxotroph cells in the presence and absence of cycloheximide. The translation rate was reduced by >80% already after 30 minutes of leucine starvation and comparable to that after cycloheximide treatment (Fig 1A). Notably, the leucine-starvation-induced eIF2α phosphorylation was completely abolished in the presence of cycloheximide (Fig 1B), demonstrating that the approach is suitable to detect a requirement for ongoing translation. In contrast, the phosphorylation of eIF2α after UVC-irradiation was not affected by blocking translation by cycloheximide prior to UVC irradiation (Fig 1C and 1D), suggesting that ongoing translation is not required for Gcn2 activation after UVC irradiation. The amount of total eIF2α did not change in response to UVC irradiation or cycloheximide treatment (S1 Fig).

It cannot be excluded that an eIF2α phosphatase is inactivated upon UVC irradiation in the presence of of cycloheximide and the increased eIF2α phosphorylation we observe is not due to increased Gcn2 activity but rather to reduced phosphatase activity. However, we think this explanation is most unlikely. Frist, we have performed in vitro kinase assays with GCN2 immunoprecipitated from unirradiated control and from UV-irradiated cells. A considerable increase in kinase activity can be clearly observed when GCN2 is immunoprecipitated from irradiated cells (Fig 1E). While this does not exclude a phosphatase being regulated, it demonstrates that the induction of GCN2 kinase activity is a major contributor to the increased eIF2α phosphorylation and supports our conclusion above that Gcn2 activation after UV irradiation does not require ongoing translation. Second, cycloheximide does not induce eIF2α phosphorylation in unirradiated control cells (Fig 1B and 1D), nor is eIF2α phosphorylation increased in cycloheximide-treated and UVC-irradiated *gcn2Δ* cells (S1 Fig). Interestingly, there is more phosphorylated eIF2α after UV irradiation in the presence of cycloheximide than in the absence (S1 Fig) suggesting that (i) a phosphatase does contribute to the regulation of eIF2α phosphorylation after UV irradiation and (ii) the regulation of the phosphatase involves translational regulation and appears to be dependent on eIF2α phosphorylation. In mammalian cells the inducible eIF2α phosphatase-targeting protein GADD34 (PPP1R15A) is expressed upon eIF2α phosphorylation and limits eIF2α phosphorylation in a negative feedback loop [27]. Although there is no obvious homologue to GADD34 in fission yeast, the increased eIF2α phosphorylation upon cycloheximide treatment after UV irradiation indicates the existence of a similar mechanism in fission yeast.

Cycloheximide was added to 100 μg/ml to inhibit translation for 10 minutes. Half the culture was irradiated with 1100 J/m^2^ as described [28] and samples were taken at the indicated times after irradiation. Note that c0 and UV0 were taken at the same time after irradiation. Leucine-starved auxotroph cells were grown in medium lacking leucine for the indicated times. At each timepoint a sample was taken to pulse label with HPG for 10 min to measure translation rates (**A, C**) and a sample was taken to extract proteins and measure eIF2α phosphorylation by immunoblotting (**B, D**). “log” refers to a sample of exponentially growing cells. On the graphs showing translation rates median fluorescence intensity from 10 000 fission yeast cells and standard deviations are shown. α-tubulin levels are shown to check even loading. **E** GCN2 was immunoprecipitated from from UV-irradiated “UV” and unirradiated control “C” cells, and incubated with extracts prepared from unirradiated *gcn2Δ* cells in kinase buffer. eIF2α phosphorylation was measured by immunoblotting. α-tubulin levels are shown to demonstrate that equal amounts of extracts from unirradiated *gcn2Δ* cells were used as substrate in the kinase assays.

### Mutating the tRNA-binding sites of Gcn2 abolishes activation in response to UVC irradiation

**Fig 2:**
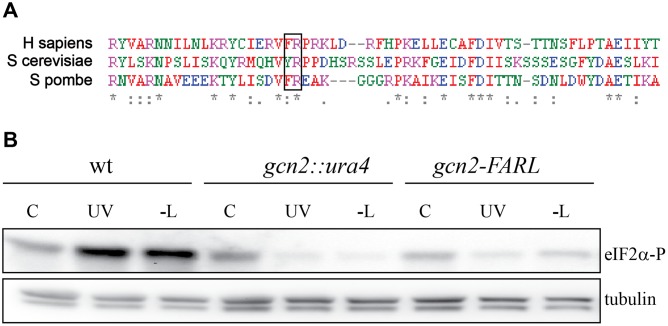
Mutating the tRNA-binding sites of Gcn2 abolishes activation in response to UVC irradiation. **A** Alignment of the HisRS-like domain of budding yeast and fission yeast Gcn2. **B** The indicated strains were irradiated with 1100 J/m^2^ and samples were taken at the indicated times after irradiation. eIF2α phosphorylation was detected by immunoblotting, α-tubulin levels are shown to check even loading.

Gcn2p contains a C-terminal domain related to histidyl-tRNA synthetases (HisRS) [29]. This domain includes residues related to the conserved motif 2 sequences that interact with the acceptor stem of the cognate tRNA in authentic class II synthetases [30]. The HisRS-like domain can bind uncharged tRNA in vitro, and mutations in the motif 2 sequence impair tRNA binding and abolish GCN2 activity in budding yeast [31]. The Y1119L R1120L motif 2 mutations rendering GCN2 inactive in budding yeast correspond to F1066A R1067L in fission yeast (Fig 2A). We introduced these two mutations in the genome of fission yeast cells and investigated whether Gcn2 can be activated by starvation. Leucine-auxotroph wild-type, *gcn2Δ*, and *gcn2*-FARL cells were starved for leucine and samples were collected at different timepoints. Phosphorylation of eIF2α was measured by immunoblotting. As expected, eIF2α was not phosphorylated after leucine starvation (Fig 2B, “-L”). eIF2 levels were not affected by either starvation or UVC irradiation in any of the strains (S2 Fig). To test whether the mutations affect Gcn2 activation after UV irradiation, we irradiated the mutant strains with UVC and monitored eIF2α phosphorylation. No induction of eFI2α phosphorylation was observed (Fig 2B, UV), suggesting that the ability of the HisRS domain to bind tRNAs is required for Gcn2 activation also after UV irradiation.

### Gcn1 is required for Gcn2 activation upon amino acid starvation in fission yeast

**Fig 3:**
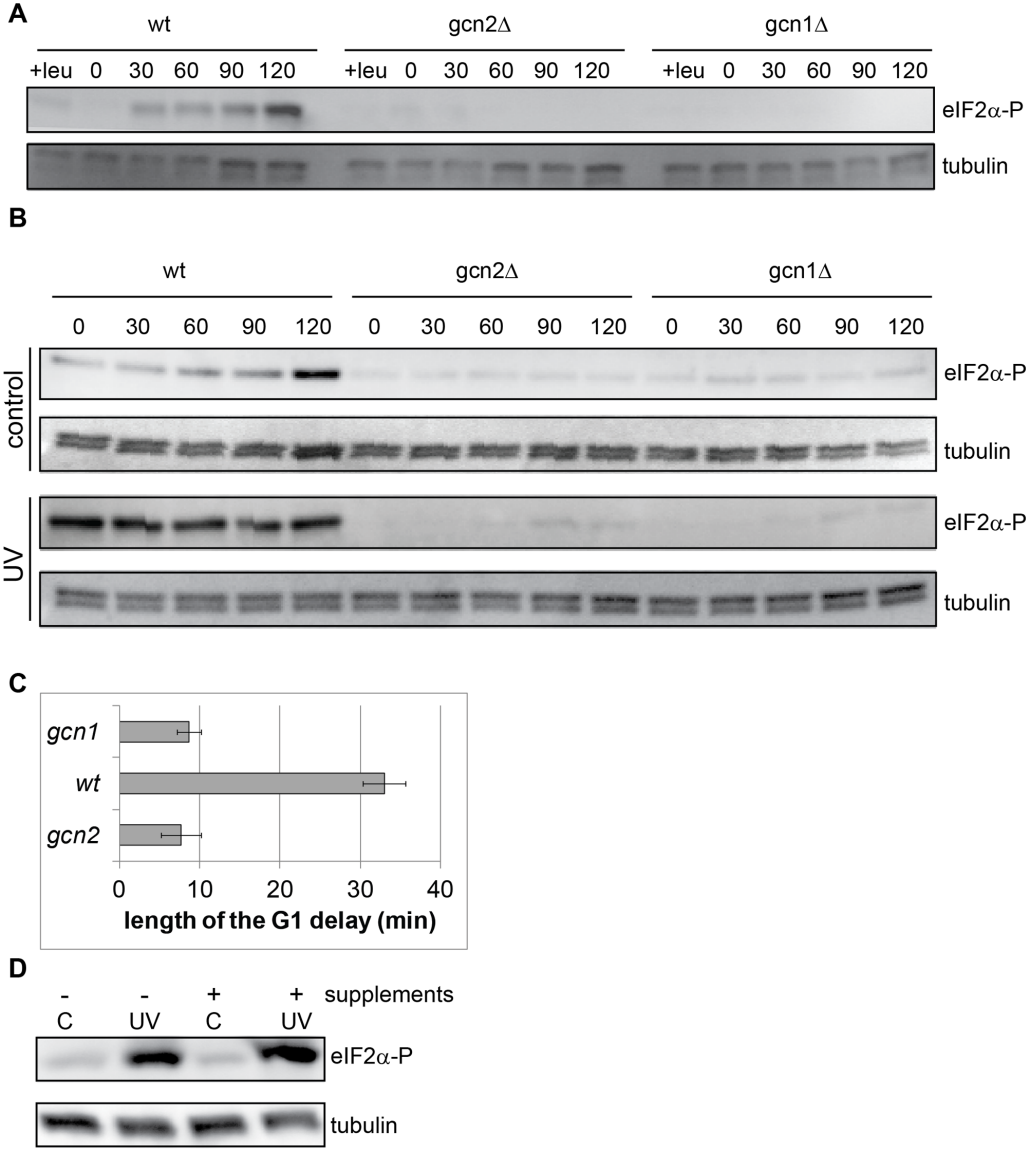
Gcn1 is required for Gcn2 activation after UVC irradiation. **A** The indicated strains were starved for leucine as described in Materials and Methods. eIF2α phosphorylation was detected by immunoblotting, α-tubulin levels are shown to check even loading. **B** eIF2α phosphorylation after UVC-irradiation in wild-type, *gcn2Δ* and *gcn1Δ* cells. Exponentially growing cells of the indicated strains were irradiated as described in Materials and methods and [23]. eIF2α phosphorylation was detected by immunoblotting, α-tubulin levels are shown to check even loading. **C** preRC loading in *gcn1Δ* cells. Cells carrying a *cdc10-M17* mutation and GFP-tagged Mcm2 were grown in EMM medium, arrested in G1 by shifting them to 36°C for 4 h, released from the G1 block and irradiated with 1100 J/m^2^ UVC. The percentage of cells containing chromatin-bound Mcm2:GFP was determined. The delay was calculated as the time difference between irradiated and unirradiated cells at reaching 70% of maximal preRC loading. **D** Prototroph wild-type cells were grown to mid-log phase in EMM medium. Supplements were added to 80 mg/l for 30 min before UV irradiation. Samples were taken immediately after irradiation. eIF2α phosphorylation was detected by immunoblotting, α-tubulin levels are shown to check even loading.

In budding yeast, two proteins have been shown to be involved in GCN2 activation after amino acid starvation, GCN1 and its cofactor GCN20. Both proteins are required to recruit GCN2 to the translating ribosome and transfer uncharged tRNAs to the HisRS domain of GCN2 [15,16,19]. To investigate whether Gcn1 is required for Gcn2 activation also in fission yeast, we deleted the putative *GCN1* homologue and investigated whether Gcn2 can be activated in *gcn1Δ* cells. Leucine was withdrawn from the medium of leucine-auxotroph wild-type, *gcn2Δ*, and *gcn1Δ* cells and samples were collected at different timepoints. Phosphorylation of eIF2α was measured by immunoblotting. In wild-type cells, phosphorylation of eIF2α occurred at 30 min after the withdrawal of leucine and persisted for at least one hour (Fig 3A). In a *gcn2Δ* mutant, eIF2α phosphorylation did not occur, showing that Gcn2 is the sole kinase responsible for the phosphorylation response after leucine starvation. In the leucine-auxotroph *gcn1Δ* strain the starvation-induced eIF2α phosphorylation was abolished (Fig 3A), demonstrating that the role of Gcn1 in the activation of Gcn2 after amino-acid starvation is conserved between budding and fission yeast.

### Gcn1 is required for Gcn2 activation after UV irradiation

If the binding of uncharged tRNAs is involved in activation of Gcn2 after other forms of stress one would expect that Gcn1 was also required. We explored whether Gcn1 is involved in the UVC-induced activation of Gcn2. Exponentially growing wild-type, *gcn2Δ*, *gcn1Δ* cells were irradiated with UVC light and samples were collected at the indicated timepoints after irradiation. UVC-irradiation-induced phosphorylation of eIF2α was clearly observed in wild-type cells and was abolished in the absence of Gcn2 (Fig 3B) consistent with previous results [23]. In the absence of Gcn1 phosphorylation of eIF2α was abolished (Fig 3B, S2 Fig), demonstrating that Gcn1 is required for the UVC-induced activation of Gcn2.

UVC-irradiation in G1 phase delays the formation of the pre-Replicative Complex (preRC) in a Gcn2-dependent manner [23] and thus delays entry into S phase. The G1 delay correlates with and possibly is caused by the phosphorylation of eIF2α [32,33]. To investigate whether Gcn1 is required for the UVC-induced G1 delay, *gcn1Δ* cells carrying a GFP-tagged Mcm2 were arrested in G1 using a *cdc10* temperature-sensitive mutation, released from the block and irradiated with UVC. Samples were taken at the indicated timepoints and the loading of the MCM complex was assessed using an in situ chromatin binding assay [23,24]. The preRC-loading delay was abolished in the irradiated *gcn1Δ* cells (Fig 3C), as previously described for *gcn2Δ* cells [23], suggesting that Gcn1 is required both for the cell-cycle delay and for Gcn2 activation after UVC-irradiation.

### Supplementing the medium with all amino acids does not prevent Gcn2 activation after UVC irradiation

The above data suggest that tRNA binding is necessary for GCN2 activation also after UVC irradiation. One plausible mechanism for the accumulation of uncharged tRNA-s is depletion of one (or a few) specific amino-acyl- tRNA due to, for example, a chemical modification or conversion. To test whether such a mechanism might be responsible for the activation of Gcn2, we supplemented the medium with all amino acids as well as adenine and uracil prior to UV irradiation. A prototroph strain was grown in EMM medium and supplements were added 30 min before irradiation. We used concentrations of supplements that were sufficient to allow growth of auxotrophic mutants. The cells were irradiated with UVC and samples were taken for immunoblotting immediately after irradiation. Providing supplements in excess in the medium did not prevent or reduce eIF2α phosphorylation (Fig 3D). These results argue against a model that Gcn2 activation after UVC irradiation is due to a specific amino-acyl- tRNA being depleted.

### Gcn1 is *required* for Gcn2 activation after H_2_O_2_- treatment

**Fig 4:**
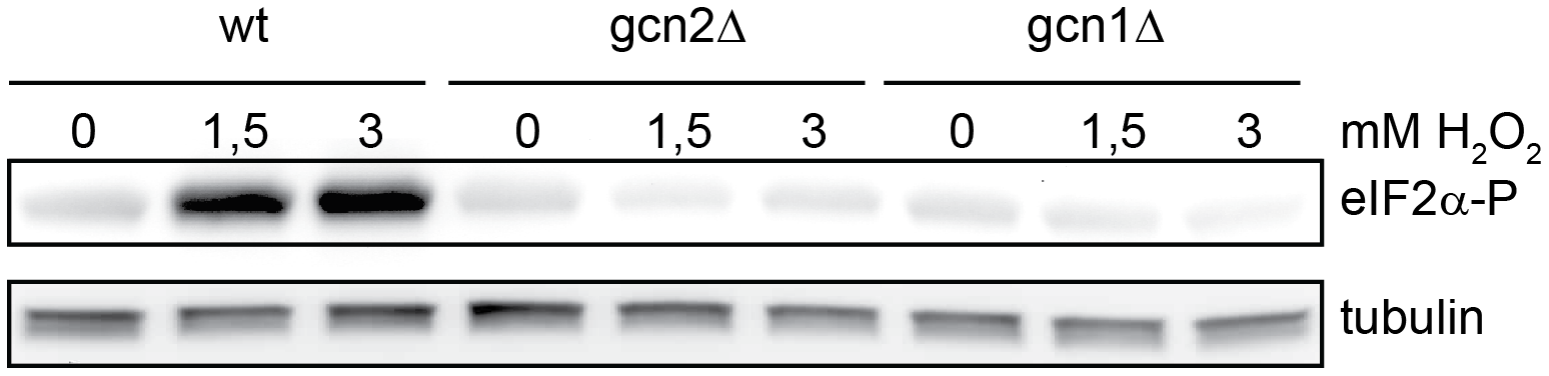
Gcn1 is required for Gcn2 activation after H_2_O_2_- treatment. eIF2α phosphorylation after H_2_O_2_ treatment in wild-type, *gcn2Δ* and *gcn1Δ* cells. The indicated strains were grown in EMM medium and treated with H_2_O_2_ at the concentrations shown, for 15 minutes. eIF2α phosphorylation was detected by immunoblotting, α-tubulin levels are shown to check even loading.

Oxidative stress has also been shown to activate Gcn2 [32,34] and again, it is not obvious how this treatment should lead to an accumulation of uncharged tRNAs. To investigate whether Gcn1 is required for the H_2_O_2_-induced activation of Gcn2, wild-type, *gcn2Δ* and *gcn1Δ* cells were grown in minimal medium, H_2_O_2_ was added to the concentrations shown and samples were collected 15 minutes after the addition of H_2_O_2_. In agreement with previous reports, phosphorylation of eIF2α was observed in response to H_2_O_2_ in wild-type cells. It should be noted that prolonged oxidative stress or high concentration of H_2_O_2_ activates another eIF2α kinase, Hri2, but the initial eIF2α phosphorylation is due to Gcn2 [34 and our unpublished results]. To study the requirement for Gcn1, we used conditions where eIF2α phosphorylation was clearly dependent on Gcn2 (Fig 4). To ensure that the cells of the different strains were exposed to the same level of oxidative stress, both the concentration of H_2_O_2_ and cell density were carefully controlled. Interestingly, eIF2α phosphorylation was abolished in the absence of Gcn1. Gcn2 protein levels were not reduced in the *gcn1Δ* mutant (S3 Fig), thus these results suggest that Gcn1 is required for Gcn2 activation also in response to oxidative stress (Fig 4).

## Discussion

The Gcn2 kinase and its regulation attract more and more attention in the field as its role in human diseases such as cancer and neurodegenerative diseases is revealed. Here we investigate mechanisms that activate Gcn2 in response to different stresses.

The molecular mechanism of GCN2 activation was first described in budding yeast after starvation. Under these circumstances GCN2 activation involves binding of uncharged tRNAs and requires the cofactor GCN1. Our findings suggest that also after UV irradiation and oxidative stress tRNA binding is required for Gcn2 activation. It should be noted that the motif 2 mutations in Gcn2 affect not only tRNA binding but also intramolecular interactions between the CTD domain and the HisRS domain, which in turn is required for activation [12]. Thus the finding that the motif 2 mutations abolish Gcn2 activation does not necessarily imply that tRNA binding is required. However, the involvement of Gcn1, thought to transfer uncharged tRNAs to the HisRS domain of GCN2 [15,16,19], strongly suggests that tRNA binding is necessary for Gcn2 activation.

Accumulation of uncharged tRNAs due to starvation is a well-described mechanism that leads to activation of Gcn2. Under these conditions it is obvious that charged tRNAs are depleted and uncharged tRNAs accumulate, but it is not clear how and why the level of charged tRNAs would be reduced in response to stresses like UVC-irradiation or oxidative stress. In fact, both of these treatments are known to reduce overall translation rates [23,35,36], making it even less likely that charged tRNAs would be depleted. Furthermore, we have shown here that ongoing translation is not required for Gcn2 activation after UV irradiation, arguing against a model that tRNA pools are depleted.

However, alternative mechanisms could be activated that would change the balance between charged and uncharged tRNAs and thus lead to Gcn2 activation. In mammalian cells after UVB irradiation nitric oxide is synthesized from Arg, leading to Arg depletion and a starvation response [37]. There is no obvious homologue to nitric oxide synthase in fission yeast, and we have shown here that supplementing the medium with all amino acids does not prevent activation of Gcn2 after UVC irradiation. Thus, it is unlikely that depletion of a specific amino acid is the reason for the UVC-induced activation of Gcn2. The activity of RNA polymerase III is inhibited in response to a number of stresses [38–40], thus it is unlikely that increased transcription of tRNAs would contribute to an increase in uncharged tRNA-s. Recent discoveries have revealed an unexpected complexity of tRNA biogenesis, as well as the role of tRNA-modifications and cleavage fragments in signaling pathways [41,42]. It is plausible that modified tRNAs or even tRNA-derived fragments are responsible for the activation of Gcn2 after UVC.

Alternatively, additional factors might be required for Gcn2 activation after stresses other than starvation. Indeed, some studies reported that Gcn2 activity can be regulated by upstream kinases. In *S*. *cerevisiae* a link between the TOR pathway and activation of Gcn2 has been shown [43]. Treating budding yeast with rapamycin, an inhibitor of the TOR kinases, leads to removal of an inhibitory phosphorylation on serine 577 of Gcn2, thought to reduce the threshold of uncharged tRNAs required for activation. However, this phosphorylation site is not conserved in fission yeast or in mammalian cells and even though inhibiting the Tor pathway can lead to increased Gcn2 activity also in fission yeast [9,44], the crosstalk between the two pathways depends on the particular stress applied [9]. In particular, Tor2 does not regulate eIF2α phosphorylation under a number of conditions, including UVC-irradiation and oxidative stress in fission yeast. Furthermore, under leucine starvation the phosphorylation of eIF2α is in fact dependent on maintained TORC1 activity rather than on TORC1 inactivation [9]. Thus it is unlikely that the Tor pathway is the master regulator of Gcn2 after UVC irradiation and oxidative stress.

Another study reported that in response to UVB-irradiation of mammalian cells Gcn2 is activated in a DNA-PK-dependent manner [45]. Thus, Gcn2 activation in human cells might be linked to DNA damage through the activity of DNA-PK, at least in response to some stresses.

It was previously reported that Gcn1 is required for Gcn2 activation after different stresses in mouse embryonic fibroblasts [46]. This conclusion is fully consistent with our results presented here. It should be noted that our experimental design is very different from that of Cambiaghi et al; here we have used loss-of function mutants in fission yeast, while they exploited dominant negative effects of overexpressing either IMPACT, a protein known to interact with Gcn1 and compete with Gcn2 for Gcn1 binding, or a portion of Gcn1^mouse^ that corresponds to the region necessary and sufficient to bind Gcn2 in budding yeast. Collectively, the two studies suggest that the requirement for tRNA binding and for Gcn1 in Gcn2 activation is a conserved feature.

In summary, it appears most likely that Gcn2 is activated by a mechanism(s) involving tRNA binding assisted by Gcn1 in response to stresses other than starvation. However, identification of the mechanisms that alter the balance between charged and uncharged tRNAs and the possible involvement of other upstream regulators requires further studies.

## Supporting information

S1 Fig UV irradiation in the presence of cycloheximide induces eIF2α phosphorylation. Wild-type and *gcn2Δ* cells were treated with 100 μg/ml cycloheximide for 10 min as indicated and UV irradiated.
(TIF)

S2 Fig eIF2α levels are not changed by the treatments. The same samples as shown in (A) Fig 2B and (B) Fig 3B were analyzed by immunoblotting using an antibody against total eIF2α.
(TIF)
