# Supplementary figures and images for: Activation of Gcn2 in response to different stresses
Source: PLoS One. 2017 Aug 3;12(8):e0182143. doi: 10.1371/journal.pone.0182143 (PMC5542535; doi:10.1371/journal.pone.0182143)

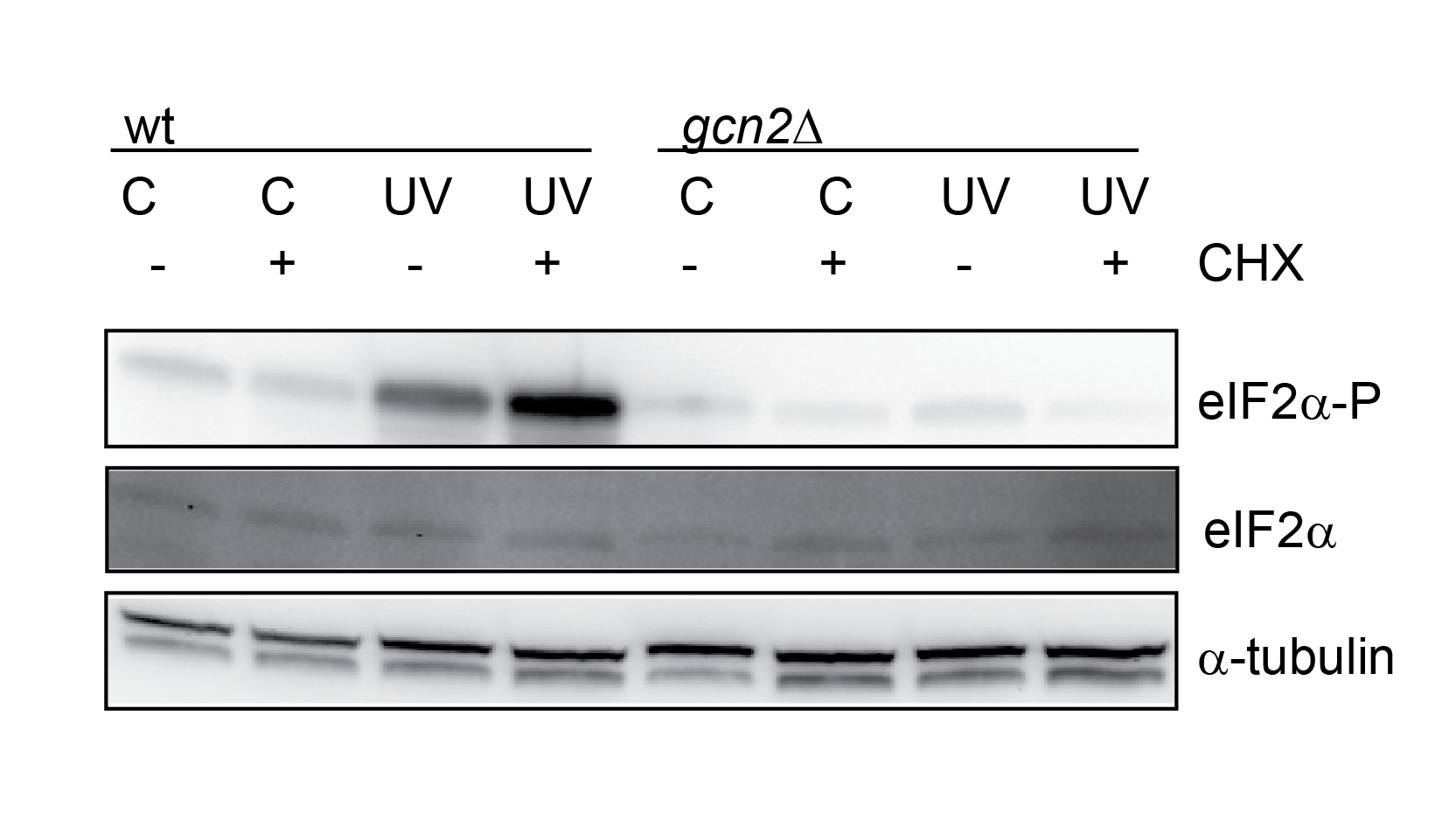

Supplement: S1 Fig — Wild-type and gcn2Δ cells were treated with 100 μg/ml cycloheximide for 10 min as indicated and UV irradiated. (TIF) [file pone.0182143.s001.tif]

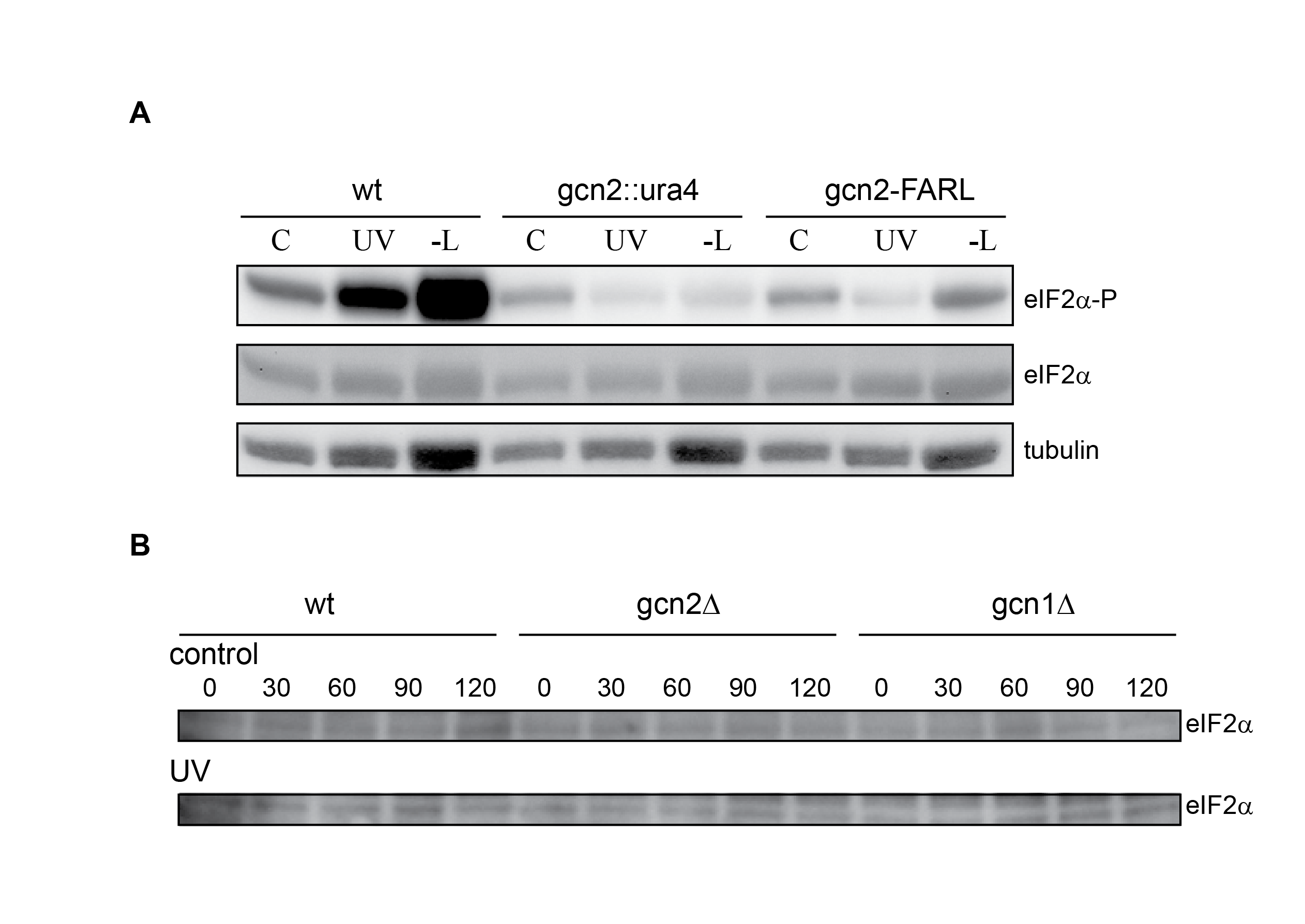

Supplement: S2 Fig — The same samples as shown in (A) Fig 2B and (B) Fig 3B were analyzed by immunoblotting using an antibody against total eIF2α. (TIF) [file pone.0182143.s002.tif]
